# Health Benefits of Nut Consumption

**DOI:** 10.3390/nu2070652

**Published:** 2010-06-24

**Authors:** Emilio Ros

**Affiliations:** Lipid Clinic, Endocrinology and Nutrition Service, Institutd’Investigacions Biomèdiques August Pii Sunyer, Hospital Clínic, Barcelona and Ciber Fisiopatología de la Obesidady Nutrición (CIBERobn), Instituto de Salud Carlos III (ISCIII), Spain; Email: eros@clinic.ub.es;Tel.: +34 93 2279393; Fax: +34 93 4537829

**Keywords:** tree nuts, peanuts, fatty acids, antioxidants, cholesterol, cardiovascular disease, diabetes, inflammation

## Abstract

Nuts (tree nuts and peanuts) are nutrient dense foods with complex matrices rich in unsaturated fatty and other bioactive compounds: high-quality vegetable protein, fiber, minerals, tocopherols, phytosterols, and phenolic compounds. By virtue of their unique composition, nuts are likely to beneficially impact health outcomes. Epidemiologic studies have associated nut consumption with a reduced incidence of coronary heart disease and gallstones in both genders and diabetes in women. Limited evidence also suggests beneficial effects on hypertension, cancer, and inflammation. Interventional studies consistently show that nut intake has a cholesterol-lowering effect, even in the context of healthy diets, and there is emerging evidence of beneficial effects on oxidative stress, inflammation, and vascular reactivity. Blood pressure, visceral adiposity and the metabolic syndrome also appear to be positively influenced by nut consumption. Thus it is clear that nuts have a beneficial impact on many cardiovascular risk factors. Contrary to expectations, epidemiologic studies and clinical trials suggest that regular nut consumption is unlikely to contribute to obesity and may even help in weight loss. Safety concerns are limited to the infrequent occurrence of nut allergy in children. In conclusion, nuts are nutrient rich foods with wide-ranging cardiovascular and metabolic benefits, which can be readily incorporated into healthy diets.

## 1. Introduction

Extensive research has been carried out on nuts and health outcomes during the last two decades since publication of a report from the pioneering Adventist Health Study showing an association of nut consumption with a lower risk of coronary heart disease (CHD) in 1992 [1], shortly followed by the seminal clinical trial of Sabaté *et al.* [2] demonstrating that a diet enriched with walnuts reduced serum cholesterol levels compared to a standard healthy diet. The interested reader will find complete information regarding research published up to 2005 on nuts and health outcomes in a recent monograph [3] and up to 2007 in the proceedings of a Symposium on Nuts and Health held at the U.S. Department of Agriculture Research Laboratory in the University of California at Davis [4]. 

By definition, tree nuts are dry fruits with one seed in which the ovary wall becomes hard at maturity. The most popular edible tree nuts are almonds (*Prunus amigdalis*), hazelnuts (*Corylus avellana*), walnuts (*Juglans regia*), and pistachios (*Pistachia vera*). Other common edible nuts are pine nuts (*Pinus pinea*), cashews (*Anacardium occidentale*), pecans (*Carya illinoiensis*), macadamias (*Macadamia integrifolia*), and Brazil nuts (*Bertholletia excelsa*). The consumer definition also includes peanuts (*Arachis hypogea*), which botanically are groundnuts or legumes but are widely identified as part of the nuts food group. In addition, peanuts have a similar nutrient profile to tree nuts [5,6]. Although chestnuts (*Castanea sativa*) are tree nuts as well, they are different from all other common nuts because of being starchier and having a different nutrient profile. For the purpose of this review, the term “nuts” includes all common tree nuts [with the exception of chestnuts] plus peanuts.

Nuts, seeds and pulses are all nutrient dense foods and have been a regular constituent of mankind’s diet since pre-agricultural times [7]. In Western countries nuts are consumed as snacks, desserts or part of a meal, and are eaten whole (fresh or roasted), in spreads (peanut butter, almond paste), as oils or hidden in commercial products, mixed dishes, sauces, pastries, ice creams and baked goods. In the last century, nut consumption in most industrialized nations followed a downward trend to become only a marginal source of energy in the daily diet, except for vegetarians and other health-conscious populations, such as Seventh Day Adventists [8,9]. However, nut consumption has increased in recent times in Western countries following both the inclusion of this food group in many guidelines for healthy eating and wide media coverage of recent evidence connecting nut consumption to a wide range of health benefits. Thus, nuts have been proposed as an important component of optimal diets for the prevention of CHD by leading experts in the field [10] and, in the summer of 2003, the US Food and Drug Administration issued a health claim for nuts because of the link between nut consumption and a reduced risk of both CHD and intermediate biomarkers, such as blood cholesterol [11]. Since then, nuts have become an indispensable component of healthy diets [12,13] and are included in the American Heart Association dietary metrics for defining ideal cardiovascular health in their recent report on setting goals for health promotion and disease reduction for 2020 [14]. 

The scientific evidence behind the proposal of nuts as cardio-protective foods stem from both epidemiological observations suggesting a consistent inverse association between the frequency of nut intake and development of CHD [reviewed in 13,15,16] and numerous short-term clinical trials showing beneficial effects of nut intake on the lipid profile [reviewed in 13,17,18,19, 20] and other intermediate markers of CHD [reviewed in 13,20,21,22]. The mechanism for these salutary effects probably lies in the synergistic interaction of the many bioactive constituents of nuts, which may all favorably influence human physiology. Thus, nuts contain high amounts of vegetable protein [5] and fat, mostly unsaturated fatty acids [6]. They are also dense in a variety of other nutrients and provide dietary fiber [23], vitamins (e.g., folic acid, niacin, tocopherols, and vitamin B6), minerals (e.g., calcium, magnesium, potassium) and many other bioactive constituents such as phytosterols [24] and phenolic compounds [25].

Contrary to expectations due to the high energy density of nuts, evidence from both epidemiological studies and clinical trials suggests that their regular consumption neither contributes to obesity nor increases the risk of developing diabetes, as reviewed [13,16,26,27]. The present review summarizes current knowledge on the expanding topic of nuts and health outcomes. First an outline of the unique nutrient content of nuts is necessary in order to better understand their health effects.

## 2. Nutrient Content of Nuts

Nuts are clearly nutrient dense foods. With the exception of chestnuts, which contain little fat, nuts have a high total fat content, ranging from 46% in cashews and pistachios to 76% in macadamia nuts, and they provide 20 to 30 kJ/g (Table 1). Thus, nuts are one of the natural plant foods richest in fat after vegetable oils. However, the fatty acid composition of nuts is beneficial because the saturated fatty acid (SFA) content is low (4-16%) and nearly half of the total fat content is made up of unsaturated fat, monounsaturated fatty acids MUFA (oleic acid) in most nuts, similar proportions of MUFA and polyunsaturated fatty acids (PUFA), mostly linoleic acid, in Brazil nuts, a predominance of PUFA over MUFA in pine nuts, and mostly PUFA, both linoleic acid and α-linolenic acid (ALA), the plant omega-3 fatty acid, in walnuts [6]. With regard to walnuts, it must be underlined that they are a whole food with the highest content in ALA of all edible plants [28]. As discussed below, the particular lipid profile of nuts in general and walnuts in particular is likely to be an important contributor to the beneficial health effects of frequent nut consumption.

Nuts are also rich sources of other bioactive macronutrients that have the potential to beneficially affect metabolic and cardiovascular outcomes. They are an excellent source of protein (approximately 25% of energy) and often have a high content of L-arginine [5]. As this amino acid is the precursor of the endogenous vasodilator, nitric oxide (NO) [29], nut intake might help improve vascular reactivity, as discussed below. Nuts also are a good source of dietary fiber, which ranges from 4 to 11 g per 100 g (Table 1), and in standard servings provide 5-10% of daily fiber requirements [23].

**Table 1:** Average nutrient composition of nuts (per 100 g).

Nuts	Energy (kJ)	Fat (g)	SFA (g)	MUFA (g)	PUFA (g)	LA (g)	ALA (g)	Protein (g)	Fiber (g)	Folate (μg)	PS (mg)
Almonds	2418	50.6	3.9	32.2	12.2	12.2	0.00	21.3	8.8	29	120
Brazil nuts (dried)	2743	66.4	15.1	24.5	20.6	20.5	0.05	14.3	8.5	22	NR
Cashews	2314	46.4	9.2	27.3	7.8	7.7	0.15	18.2	5.9	25	158
Hazelnuts	2629	60.8	4.5	45.7	7.9	7.8	0.09	15.0	10.4	113	96
Macadamia nuts	3004	75.8	12.1	58.9	1.5	1.3	0.21	7.9	6.0	11	116
Peanuts	2220	49.2	6.8	24.4	15.6	15.6	0.00	25.8	8.5	145	220
Pecans	2889	72.0	6.2	40.8	21.6	20.6	1.00	9.2	8.4	22	102
Pine nuts (dried)	2816	68.4	4.9	18.8	34.1	33.2	0.16	13.7	3.7	34	141
Pistachios	2332	44.4	5.4	23.3	13.5	13.2	0.25	20.6	9.0	51	214
Walnuts	2738	65.2	6.1	8.9	47.2	38.1	9.08	15.2	6.4	98	72

Data for raw nuts, except where specified. SFA, saturated fatty acids; MUFA, monounsaturated fatty acids; PUFA, polyunsaturated fatty acids; LA, linoleic acid; ALA, α-linolenic acid; PS, plant sterols; NR, not reported. Source: US Department of Agriculture Nutrient Data Base at: http://www.nal.usda.gov/fnic/cgi-bin/nut_search.pl (Accessed on 26 April 2010).

Among the constituents of nuts there are significant amounts of essential micronutrients that are associated with an improved health status when consumed at doses beyond those necessary to prevent deficiency states. Nuts contain sizeable amounts of folate (Table 1) [24], a B-vitamin necessary for normal cellular function that plays an important role in detoxifying homocysteine, a sulfur-containing amino acid with atherothrombotic properties that accumulates in plasma when folate status is subnormal [30]. Nuts are also rich sources of antioxidant vitamins (e.g., tocopherols) and phenolic compounds, necessary to protect the germ from oxidative stress and preserve the reproductive potential of the seed, but also bioavailable after consumption and capable of providing a significant antioxidant load [25]. Almonds in particular are especially rich in α-tocopherol, while walnuts contain significant amounts of its isomer γ-tocopherol, which has been investigated much less than α-tocopherol, but is increasingly recognized as a relevant antiatherogenic molecule [31]. Remarkably, in all nuts most of the antioxidants are located in the pellicle or outer soft shell, as shown for almonds [32,33] and peanuts [34], and 50% or more of them are lost when the skin is removed [25]. Bleaching of nuts when the hard shells are cracked, as it occurs naturally in pistachios, also destroys most of the antioxidants [35]. Interestingly, roasting of almonds preserves the phenolic compounds better than blanching [36]. These facts, rarely taken into consideration in prior studies with nuts, should not be overlooked when giving advice on nut intake in healthy diets. Walnuts are an exception because they are almost always consumed as a raw, unpeeled product.

Nuts are cholesterol-free, but their fatty fraction contains sizeable amounts of chemically related noncholesterol sterols belonging to a heterogeneous group of compounds known as plant sterols or phytosterols (Table 1) [24]. They are non-nutritive components of all plants that play an important structural role in membranes, where they serve to stabilize phospholipid bilayers just as cholesterol does in animal cell membranes [37]. Phytosterols interfere with cholesterol absorption and thus help lower blood cholesterol when present in sufficient amounts in the intestinal lumen. The mechanism of action of phytosterols has been linked to their hydrophobicity, which is higher than cholesterol because of a bulkier hydrocarbon molecule and entails a higher affinity for micelles than has cholesterol. Consequently, cholesterol is displaced from micelles and the amount available for absorption is limited [36]. In all probability the phytosterol content of nuts contributes to their cholesterol-lowering effect (see below).

Compared to other common foods, nuts have an optimal nutritional density with respect to healthy minerals, such as calcium, magnesium, and potassium. Like that of most vegetables, the sodium content of raw or roasted but otherwise unprocessed nuts is very low, ranging from undetectable in hazelnuts to 18 mg/100 g in peanuts (Table 2) [24]. A high intake of calcium, magnesium and potassium, together with a low sodium intake, is associated with protection against bone demineralization, arterial hypertension, insulin resistance, and overall cardiovascular risk [39]. Obviously, the advantage of the low sodium content of nuts is lost if they are consumed as a salted product.

In summary, the macronutrient, micronutrient and non-nutrient components of nuts shown in Table 1 and Table 2 have all been documented to contribute to a reduced risk of CHD and related metabolic disturbances. For these reasons, whole raw, unpeeled and otherwise unprocessed nuts may be considered as natural health capsules, where the whole is always better than the parts [40].****

**Table 2:** Calcium, magnesium, sodium and potassium content of nuts and other foods in mg/100 g of edible portion.

Nuts	Calcium	Magnesium	Sodium	Potassium
Almonds	248	275	1	728
Brazil nuts	160	376	3	659
Cashew nuts	37	292	12	660
Hazelnuts	114	163	0	680
Macadamia nuts	85	130	5	368
Peanuts	92	168	18	705
Pecans	70	121	0	410
Pine nuts	16	251	2	597
Pistachios	107	121	1	1025
Walnuts	98	158	2	441
**Other foods**				
Apples (with skin)	6	5	1	107
Bananas	5	27	1	358
Beans (white, cooked)	90	63	6	561
Broccoli (cooked)	40	21	41	293
Cheese (cheddar)	721	28	621	98
Chickpeas (cooked)	49	48	7	291
Ham	24	22	1304	287
Lettuce (romaine)	33	14	8	247
Milk	113	10	40	143
Rice (white, cooked)	10	38	1	35
Sardines (canned in oil)	382	39	505	397
Spinach (cooked)	136	87	70	466
Tomato	10	11	5	237
Veal (cooked)	22	26	87	325
Wheat bread	72	86	527	252

Source: US Department of Agriculture Nutrient Data Base at: http://www.nal.usda.gov/fnic/cgi-bin/nut_search.pl (Accessed on 26 April 2010).

## 3. Epidemiological Studies of Nut Consumption and Health Outcomes

### 3.1. Nut Consumption and CHD Risk

**Figure 1:**
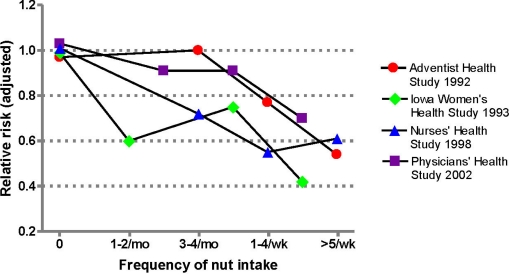
Results of prospective studies of nut consumption and risk of death from coronary heart disease.

Four prospective studies conducted in the US have reported a beneficial effect of nut consumption on CHD incidence after follow-up ranging from six to 18 years of large cohorts of previously healthy subjects [1,41,42,43]. A pooled analysis of these studies shows that subjects in the highest intake group for nut consumption had a 37% reduction in multivariable-adjusted risk of fatal CHD [15]. The combined relative risk for total CHD mortality derived from the comparison of rates between the highest and lowest frequency of nut intake in all four studies was 0.63 [95% confidence interval [CI], 0.51 to 0.83]. Importantly, a dose-response relationship between nut consumption and reduced CHD mortality rates was reported for all four studies, strengthening the causal link (Figure 1). Of particular note are the results of the Physicians’ Health Study [43], where the inverse association between nut consumption and total CHD mortality was primarily due to a reduction in sudden cardiac death. Compared with men who rarely or never consumed nuts, those who consumed nuts two or more times per week had a 47% reduced risk of sudden cardiac death (relative risk, 0.53; CI, 0.30 to 0.92).

In the Nurses’ Health Study [41], nut intake could be subdivided between all tree nuts and peanuts and peanut butter. Consumption of peanut products was also associated with a decreased relative risk of CHD. Subjects who consumed peanuts two or more times per week had a relative risk of CHD of 0.66 (CI, 0.46 to 0.94), while for other nuts the relative risk for consumption of two or more times per week was 0.79 (CI, 0.50 to 1.25). A recent report of the same study concerning a subgroup of 6309 women with type-2 diabetes at baseline shows similar findings, as consumption of at least five servings per week of nuts or peanut butter (serving size, 28 g [1 ounce] for nuts and 16 g [1 tablespoon] for peanut butter) was significantly associated with a lower risk of cardiovascular disease, with a relative risk of 0.56 (CI, 0.36 to 0.89) [44].

It must be underlined that in all these studies [1,41,42,43,44] the protective effect of nut consumption on total CHD or sudden cardiac death was independent of gender, age, body mass index, alcohol use, other nutritional characteristics or presence of cardiovascular risk factors. The dose-relationship between nut intake and incident CHD translates into an average 8.3 reduction for each weekly serving of nuts [15]. The consistency of findings in all studies points to a causal association between nut consumption and reduced CHD, indicating that nuts possibly are one of the most cardioprotective foods in the habitual diet.

Since inflammation is a key process in atherogenesis, one mechanism by which nut consumption may decrease CHD risk is by improving inflammatory status, which can be ascertained from levels of circulating inflammatory markers. Three cross-sectional studies have investigated nut consumption in relation to circulating inflammatory biomarkers [45,46,47]. In an analysis of data from nearly 6000 participants in the Multi-Ethnic Study of Atherosclerosis (MESA) [45], consumption of nuts and seeds was inversely associated with levels of inflammatory markers, C-reactive protein (CRP), interleukin-6 (IL-6) and fibrinogen. Another study of 987 diabetic women from the prospective Nurses’ Health Study [46] showed a direct association between nut consumption and increased plasma levels of adiponectin, an adipose tissue-secreted cytokine with antiinflammatory and antiatherosclerotic properties. The third study was carried out in 772 older subjects at high risk for CHD living in Spain for the purpose of assessing adherence to the Mediterranean dietary pattern and its food components in relation to levels of soluble inflammatory markers. Adjusted mean serum levels of intercellular adhesion molecule-1 (ICAM-1), but not those of CRP or IL-6, decreased across increasing tertiles of nut consumption [47]. Thus, increasing epidemiologic evidence links frequent consumption of nuts to a reduced inflammatory status, which might help explain their cardio protective properties.

### 3.2. Nut Consumption and Risk of Type-2 Diabetes

In addition to cardiac outcomes, the Nurses’ Health Study also ascertained the incidence of type-2 diabetes, a major risk factor for CHD, by frequency of nut and peanut butter intake during a 16-year follow-up [48]. Nut consumption was inversely associated with risk of type-2 diabetes after multivariate adjustment for traditional risk factors, with relative risks across categories of nut consumption (never/almost never, <once/week, 1-4 times/week, and >4 times/week) for a 28 g serving of 1.0, 0.92 (CI, 0.85 to 1.00), 0.84 (CI, 0.76 to 0.93), and 0.73 (CI, 0.60 to 0.89). Considering only lean women (BMI< 25 kg/m^2^), a 45% risk reduction was observed in those consuming nuts five times or more per week. Consumption of peanut butter was also inversely associated with type-2 diabetes with an adjusted relative risk of 0.79 (CI, 0.68 to 0.91) in women consuming peanut butter more than four times a week (equivalent to≥15 ounces of peanuts per week) compared with those who never or almost never ate peanut butter. 

However, in the Iowa Women’s Health Study [49] the association between nut consumption and diabetes risk was less clear. In the 11 years of follow up, the postmenopausal women who ate nuts often had no reduced risk of diabetes compared to those who ate nuts occasionally after adjusting for multiple confounders. These negative findings might have been due to over adjustment for nutrients that mediate in part the protective effect of nuts, such as fiber and unsaturated fatty acids, because when adjusting for age only a significant 18% reduction in relative risk was observed between the highest and lowest categories of peanut butter consumption. To ascertain whether menopausal status influenced the association between nut consumption and diabetes risk, in response to the analysis of the Iowa Women’s Health Study authors of the Nurse’s Health Study report performed additional analysis stratifying by menopausal status, and the inverse association of nut consumption and diabetes risk changed little. Among premenopausal women the multivariate relative risk was 0.67 (CI, 0.46 to 0.97) comparing those who ate nuts five times or more times a week with those who never or almost never ate nuts. Among postmenopausal women the corresponding relative risk was similar at 0.73 (CI, 0.57 to 0.95) [49]. A subsequent report from a Chinese cohort of nearly 64,000 women followed up for 4.6 years also suggests a protective effect of nuts on diabetes risk [50]. This study showed an adjusted 20% risk reduction between the lowest quintile (0.1 g) and upper quintile (3.1 g) of daily peanut consumption. 

**Figure 2:**
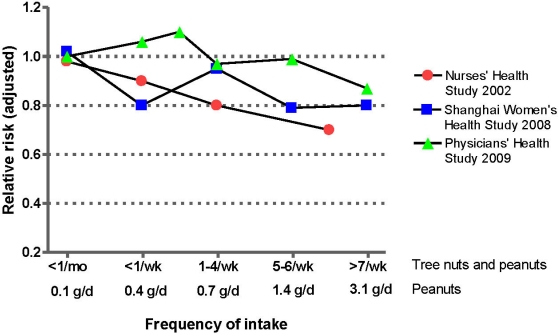
Results of prospective studies of nut consumption and risk of diabetes. The two US studies considered the frequency of consumption of all nuts, including peanuts, while the Chinese study considered exclusively quintiles of peanut consumption in grams/day.

At odds with the findings in women, a recent report from the Physicians’ Health Study [51] suggests no protective effect of nut consumption on diabetes risk in men. In this study 20,224 male participants were followed for an average of 19 years. Adjusted hazard ratios for development of diabetes ranged from 1.06 (CI, 0.93 to 1.20) for men consuming less than one serving of nuts per week to 0.87 (CI, 0.61 to 1.24) for those eating at least a daily serving of nuts, and the results were similar in lean or overweight/obese participants. Figure 2 illustrates the findings of the main prospective studies relating nut consumption to the risk of developing type-2 diabetes. In summary, regular consumption of nuts is clearly beneficial for CHD risk, but confirmation of any protective role on diabetes risk must await further studies.

### 3.3. Nut Consumption and other Health Outcomes

Two prospective studies have assessed the frequency of nut consumption in relation to incident hypertension, with discordant results [52,53]. In a cohort of 15,966 participants in the Physicians’ Health Study [52] who were free of hypertension at baseline and had 237,585 person-years of follow up, adjusted hazard ratios for hypertension ranged from 0.97 (CI, 0.91 to 1.03) for nut consumption 1‑2 times per month to 0.82 (CI, 0.71 to 0.94) for nut consumption of seven or more times per week. In a secondary analysis stratified by BMI, there was an inverse relationship between nut intake and hypertension in lean subjects but not in those who were overweight or obese at baseline. These results must be taken with caution, however, because salt intake and changes in weight, two major factors that influence the risk of hypertension, were not accounted for in this study. The second study, which involved 9919 Spanish university graduates followed-up for a median of 4.3 years in the SUN cohort [53], found no association between nut consumption and incidence of hypertension after adjusting for several confounders, including exposure to salt and weight changes during follow-up. The hazard ratio for the highest *versus* lowest nut consumption category was 0.77 (CI, 0.46 to 1.30) in this relatively young sample of well educated adults at little baseline risk for hypertension, thus a larger sample and longer duration of follow-up might have provided a better level of evidence. In summary, limited epidemiologic data provide only circumstantial evidence for a protective effect of nut consumption on development of hypertension. The fact that nuts are often eaten with salt from snack packs is an added source of confusion in the relationship between nut consumption and hypertension.

The incidence of two major complications of hypertension, stroke and heart failure, was unrelated to the frequency of nut consumption in recent reports from the prospective Physicians’ Health Study [54,55]. Regarding stroke, while no association with total or ischemic stroke was observed, there was a suggestive non-linear relation between nut intake and hemorrhagic stroke: compared to subjects who did not consume nuts, adjusted hazard ratios for hemorrhagic stroke for subjects consuming nuts <1, 1, 2-4, 5-6, and 7 or more times per week were 1.13 (CI, 0.78 to 1.62), 1.05 (CI, 0.70 to 1.58), 0.49 (CI, 0.27 to 0.89), 1.50 (CI, 0.79 to 2.84), and 1.84 (CI, 0.95 to 3.57), respectively (p for quadratic trend 0.12) [50]. However, there were a limited number of hemorrhagic strokes in the highest categories of nut consumption, thus further studies are clearly warranted to confirm or discard this improbable adverse effect of nuts.

Some bioactive constituents of nuts, such as tocopherols, phytosterols, folic acid, selenium, and magnesium, are purported to have antioxidant, antiinflammatory or anticarcinogenetic properties [24], a reason why a protective effect of nut consumption on cancer risk might be hypothesized [56]. Old epidemiological evidence of the role of nut consumption on cancer incidence was inconclusive [57,58,59]. More recent reports support a preventive role, although limited to women [56,57,58,59]. A small case-control study in Greek women [60] suggests that a diet rich in nuts, seeds and pulses reduces the risk of endometrial cancer by 27% compared to infrequent consumption of such foods. Results from the large EPIC study [61] showed no relation between higher intake of nuts and seeds and risk of colorectal cancers in the whole cohort or in men alone, but an inverse association was detected in women between the highest quintile of nut consumption (>6.2 g/day) and the lowest quintile (non‑consumers), with an adjusted odds ratio of 0.69 (CI, 0.50 to 0.95). A gender discrepancy in the risk of colorectal cancer associated with peanut consumption was also reported from a population-based cohort study of approximately 24,000 people in Taiwan [62]. This study showed that women consuming peanuts had a remarkable risk reduction of 58% compared to non-consumers. However, the protective effect was not observed in men. A small clinical study in men at risk for prostate cancer showed increased serum γ-tocopherol and a trend towards an increase in the ratio of free prostate specific antigen (PSA): total PSA after eight weeks of a diet supplemented with 75 g walnuts per day compared with a control diet [63]. Two recent experimental studies using human cancer cell lines [64] and a mice model of human breast cancer [65] suggest an antiproliferative effect of walnuts. Clearly, more research is necessary on the important topic of nuts and cancer.

Again because of the richness of nuts in bioactive components, particularly unsaturated fatty acids, fiber, and minerals, a protective effect of nut intake on gallstone disease is biologically plausible. Two separate studies by the same authors, each on different populations, examined the relationship between frequency of nut intake and gallstone disease risk. After following 80,718 women for 20 years, the Nurses’ Health Study [66] showed that frequent nut consumers (≥5/week) had a 25% reduced risk of cholecystectomy compared to non-consumers. Similar findings were observed among nearly 43,000 men in the Health Professional’s Follow-up study [67]. During 457,305 person-years of follow-up, men who consumed 5 or more servings of nuts per week showed a risk of developing clinical gallstone disease that was 30% lower compared to those who rarely or never ate nuts. The results of the two studies suggest that frequent nut consumption is equally protective of gallstone disease in men and women.

Finally, patients with diverticular disease of the colon are frequently advised to avoid eating nuts and seeds to reduce the risk of complications but there is little evidence to support this recommendation, a reason why investigators from the Health Professionals Follow-up Study [68] evaluated the frequency of nut consumption in relation to new diagnoses of diverticular disease and its complications during 18 years of follow-up in 47,228 men. The results showed an inverse association between nut consumption and the risk of diverticulitis, with a multivariate hazard ratio for men with the highest intake compared with those with the lowest intake of 0.80 (CI, 0.63 to 1.01). No associations were seen between nut consumption and diverticular bleeding or uncomplicated diverticulosis. Clearly there are no reasons to recommend avoiding nuts to prevent diverticular complications.

## 4. Nut Feeding Trials with Outcomes on Cardiovascular Risk Factors

The epidemiologic evidence reporting benefits of nut consumption on CHD risk was the impetus for clinical studies designed to assess the effects on cardiovascular risk factors and begin to understand the underlying mechanisms that explained the observational data. Most clinical studies with nuts have been short-term and have compared diets supplemented with nuts with control diets for outcomes on blood lipid changes in healthy subjects or patients with moderate hypercholesterolemia. There have been fewer studies with nuts in patients with obesity, the metabolic syndrome, or type-2 diabetes investigating insulin sensitivity or glycemic control besides the lipid profile. More recent clinical trials have dealt with intermediate risk markers, such as blood pressure, oxidation biomarkers, antioxidant defenses and oxidative modification of lipids or DNA, and inflammation status. Some studies have focused on the relevant question of whether unrestricted nut intake leads to weight changes. Long-term studies targeting effects of nut consumption on metabolic syndrome, diabetes, CHD events and risk for chronic degenerative diseases are underway.

### 4.1. Effects of Nuts on the Lipid Profile

The first clinical trial using nuts was the Loma Linda University walnut study, published in 1993 [2]. In this landmark study, a cholesterol-lowering diet that provided 20% of energy from walnuts and 31% of energy from fat, of which 6% came from SFA and 16% from PUFA, was compared to a standard Step-I diet that provided 30% of energy from fat, of which 10% was from SFA and 10% from PUFA. Total cholesterol and LDL cholesterol decreased significantly by 12% and 18%, respectively in the healthy subjects studied. Since then, over 40 clinical studies have been conducted assessing the effects of nut-enriched diets *versus* isoenergetic, usually healthy comparator diets, on serum lipids and lipoproteins, as reviewed up to December 2004 in a pooled analysis of 25 intervention trials using different nut types [69] and through May 2008 in a meta-analysis of 13 feeding studies with walnuts [70].

The nuts most frequently studied have been almonds and walnuts. Some feeding trials used peanuts, pecans, macadamia nuts, hazelnuts, pistachios, cashews, and Brazil nuts. To date there have been no clinical studies with pine nuts. In the feeding trials the nut-supplemented diets were compared to various control diets: low in total fat and high in carbohydrate; high in SFA; a Mediterranean diet; the Japanese diet; or subjects’ usual diet. Although the degree of dietary control was variable, ranging from being tightly controlled (*i.e.*, all foods provided by the investigators) to simply providing dietary advice to free-living participants eating on their own, the results have been consistent in showing a cholesterol-lowering effect of regular nut intake, usually without any significant effect on triglycerides or HDL cholesterol [2,13,17,18,19,20,69,70]. 

**Figure 3:**
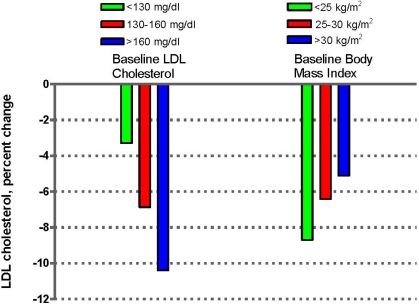
LDL-cholesterol response to nut feeding by baseline LDL-cholesterol level and BMI. Data from a pooled study of 25 nut feeding trials (adapted from ref. 69).

Recently the findings of a pooled analysis of 1,284 observations contributed by 583 unique participants from 25 clinical studies performed with different nuts, including peanuts, and conducted in seven different countries have been reported [69]. The results show a dose-response cholesterol-lowering effect and indicate that, for an average daily intake of 67 g of nuts (roughly equivalent to 20% of energy), the mean estimated reductions of total cholesterol and LDL-cholesterol were 11 mg/dL (5%) and 10 mg/dL (7%), respectively. Nuts had no significant effect on HDL-cholesterol or triglycerides, except in participants with serum triglycerides >150 mg/dL, in whom a significant 10.2 mg/dL reduction was observed. Importantly, the lipid effects of nuts were dose-related, similar by gender and across all age groups, and independent of the type of nut tested. The statistical power of this pooled analysis also allowed detection of differential responses by baseline LDL-cholesterol level and BMI. The estimated cholesterol lowering effect of nuts was greater for participants with higher initial values of LDL-cholesterol and, noticeably, for those with lower baseline BMI (Figure 3).

A recent meta-analysis [70] examined 13 clinical trials involving 365 participants who received diets supplemented with walnuts accounting for 5% to 25% of total energy and lasting 4-24 weeks. When compared with control diets, walnut-rich diets resulted in a significantly greater decrease in total and LDL-cholesterol concentrations, with weighted mean decreases of 10.3 and 9.2 mg/dL, respectively. The overall result indicated that the walnut diets compared with the control diets were associated with a 6.7% greater decrease in LDL-cholesterol concentration, which concurs with the mean 7% decrease reported with various nut types in the pooled analysis [69]. HDL-cholesterol and triglycerides were not significantly affected by walnut diets more than by control diets.

Recent well controlled intervention studies with walnuts [71,72,73,74], almonds [75], hazelnuts [76], pistachios [77], macadamias [78], and peanuts [79] showed LDL-cholesterol reductions ranging from 4% to 11% *versus* comparator diets, confirming the cholesterol-lowering efficacy of various nut types. A Mediterranean diet supplemented with 30 g of mixed nuts (walnuts, almonds and hazelnuts) per day also showed beneficial effects on the lipid profile compared with advice on a low-fat diet in diabetic and non diabetic participants in the PREDIMED study, a randomized trial of dietary intervention for the primary prevention of cardiovascular disease [80]. Of note, two randomized trials that used cashews or walnuts [81] and mixed nuts [82] compared to control diets in obese patients with the metabolic syndrome failed to show the predictable cholesterol lowering-effect, which supports the findings of the pooled analysis [69] (Figure 3) regarding the inverse association between cholesterol responses to nut feeding and BMI. There may be a mechanistic explanation for decreased lipid responsiveness to dietary intervention in patients with the metabolic syndrome. Studies have shown that the LDL cholesterol response to diets low in SFA [83] or to egg feeding as dietary cholesterol challenge [84] are blunted in obese, insulin-resistant subjects compared with lean, insulin-sensitive individuals. Prior studies had shown that higher BMI is associated with decreased LDL-cholesterol responses to hypolipidemic diets [85,86,87]. High cholesterol synthesis and reduced intestinal cholesterol absorption in insulin-resistant states [88,89] might explain these findings, as an enhanced cholesterol flux through the liver will down-regulate LDL receptors and make them refractory to additional regulation by dietary fatty acid changes, while a decreased cholesterol flux though enterocytes would lessen both the cholesterol-raising response to dietary cholesterol and the cholesterol-lowering effect of plant sterols. Nuts are rich in plant sterols, which are likely to contribute to their cholesterol lowering effect [24], but this would be less operative when cholesterol absorption is low.

Nut consumption decreases total and LDL-cholesterol, but the response does not completely agree with that expected on the basis of the dietary fatty acid and cholesterol exchange between nut diets and control diets [90]. As discussed [19], the decrease in LDL-cholesterol by nut diets is greater than predicted in most studies. This suggests that nut constituents other than fatty acids, such as fiber and/or phytosterols [23,24] are also bioactive in lowering blood cholesterol. Recently suggestive evidence has been provided that phytosterols in nuts relate to the LDL-cholesterol response observed after their consumption [91].

### 4.2. Nuts, Insulin resistance and Glycemic Control

Some interventional studies have examined the effects of nut-enriched diets on glycemic control in diabetic patients and insulin sensitivity in insulin-resistant states. Nuts had no discernible effect on fasting or postprandial glucose and hemoglobin A1C in patients with diabetes [73,92,93,94]. Changes in insulin sensitivity in response to nut diets have been inconsistent. No effects were seen in feeding studies of healthy subjects [92], hyperlipidemic patients [95], or patients with insulin-resistant states, such as obesity [96] or the metabolic syndrome [97,98]. Two recent small studies, however, found reduced insulin levels in patients with metabolic syndrome [82] and diabetes [73] after nut feeding. The three-month report of the larger PREDIMED study [80] also showed that the Mediterranean diet enriched with nuts was associated with improved insulin sensitivity and fasting glucose levels in non diabetic and diabetic participants, respectively. Finally, two studies from the same group [99,100] reported reduced postprandial glucose and insulin excursions after almond meals compared with those elicited after meals containing carbohydrates with a high glycemic index. Thus, in spite of their high energy and fat load, nuts do not worsen and may even improve metabolic control or insulin sensitivity in insulin-resistant states, but more evidence is necessary.

### 4.3. Effects of Nuts on Emerging Cardiovascular Risk Factors

By virtue of their unique fat and non-fat composition, nuts are likely to affect markers of atherogenesis other than the lipid profile or carbohydrate metabolism. More recently, the effects of nuts on novel CHD risk factors have been evaluated, including oxidative stress, inflammation and vascular reactivity, as reviewed [13,20,21,22]. The emerging picture is that frequent nut consumption has beneficial effects on cardiovascular risk factors beyond well-established cholesterol lowering.

#### 4.3.1. Oxidation

Nuts are important sources of tocopherols and phenolic compounds with potent antioxidant effects, as shown by reduction of lipid peroxidation or oxidative DNA damage with nut extracts in studies *in vitro* and the beneficial effects of nut intake on lipid oxidation, antioxidant enzyme activity, and formation of cholesterol oxidation products in both acute and chronic experimental animal studies [22,25]. Recently, walnuts were shown to contain substantial amounts of melatonin, which contributed a significant antioxidant effect in an experimental rat model [101]. In addition, because an important fraction of the fat contained in most nuts is made of MUFA, which is not a substrate for oxidation, enrichment of lipoprotein lipids with these fatty acids after nut consumption might decrease their susceptibility to oxidation. Nuts, especially walnuts, are also good sources of PUFA, and double bonds in the molecular structures of these fatty acids are preferred initiation sites for oxidation reactions [102]. Consequently, detrimental changes of lipoprotein oxidation might be expected to occur after walnut consumption unless counteracted by endogenous antioxidants in these nuts.

Oxidative markers after feeding of MUFA-rich nuts, predominantly almonds, but also hazelnuts, peanuts, pistachios, macadamia nuts, cashews, pecans, and Brazil nuts, have been examined in several randomized feeding studies, usually of small size and lasting from three to eight weeks, as comprehensively reviewed up to 2008 [22]. Biomarkers of oxidation were secondary outcomes in most of these studies, which showed inconsistent results, with either reduced or unchanged oxidation, but in no case worse oxidative status, compared with various control diets. Several feeding studies of similar characteristics have assessed oxidative biomarkers after consumption of diets supplemented with PUFA-rich walnuts *versus* other healthy diets [reviewed in 21,22]. In general, there were no between-diet differences in oxidative status, probably because as discussed antioxidants present in walnuts likely prevented the potentially adverse effects of increasing the PUFA content of biological membranes.

Four recent studies have assessed the acute effects of meals enriched with nuts on postprandial oxidation in comparison with nut-free meals [99,103,104,105]. Results have again been mixed, as two studies using walnuts [103] and almonds [104] had no discernible effect on oxidation biomarkers, while one study with almonds [99] and another study examining both almond and walnut meals [105] showed beneficial effects on postprandial oxidative stress.

In a recent parallel feeding trial with higher statistical power than usual clinical studies with nuts, the PREDIMED study [106], a Mediterranean diet enriched with 30 g mixed nuts (half of it walnuts, the rest almonds and hazelnuts) given daily for 12 weeks to subjects at high cardiovascular risk resulted in a lower oxidized LDL level compared with the control diet. Conversely, a smaller study using the same mixed-nut diet against a similar healthy diet without nuts for 12 weeks in patients with metabolic syndrome failed to show any between-diet differences in oxidized LDL and other oxidation biomarkers, except for reduced DNA damage with the nut diet [107].

In summary, available evidence from clinical studies suggests that MUFA-rich nuts may moderately improve oxidative status, while PUFA-rich nuts (walnuts) have a neutral or slightly beneficial effect, but no studies have shown that frequent nut consumption reduces antioxidant defenses.

#### 4.3.2. Inflammation

The high content of phenolic compounds in nuts, particularly in the pellicle, might anticipate an antiinflammatory effect of frequent nut consumption [108], as suggested in cross-sectional studies [43,44,45]. Walnuts could be predicted to be more antiinflammatory than other nuts for two reasons. First, as discussed, walnuts are the only nuts that contain substantial amounts of ALA, which is described as one of the more anti-inflammatory fatty acids [109,110]. And second, walnuts are also particularly rich in the phenolic compound ellagic acid, which has shown potent anti-inflammatory properties in experimental studies [111,112]. Nevertheless, plasma levels of CRP, a standard measure of systemic low-grade inflammation, were usually unaffected in controlled feeding trials with almonds, walnuts, or mixed nuts, as reviewed up to 2008 [21]. On the other hand, other inflammatory mediators such as plasma levels of ICAM-1, vascular cell adhesion molecule [VCAM]-1, or IL-6 decreased after nut diets in two studies [80,113]. It must be noted that inflammatory biomarkers were always secondary outcomes of nut-feeding trials, thus problems of statistical power to detect significant changes are a problem. The same can be said of a recent small interventional study using diets enriched with two doses of almonds *versus* a healthy, nut-free diet [114]. However, in this study almond diets were superior to the control diet to reduce circulating CRP and also E-selectin, another potent inflammatory cytokine, but not IL-6.

Two recent studies have examined the acute effects of walnut-rich meals on postprandial inflammation [103,115]. The study of Cortés *et al.* [103] compared high-SFA meals supplemented with either walnuts or olive oil on postprandial events in healthy and hypercholesterolemic subjects and found that postprandial rises of inflammatory markers were similarly blunted after the two meals, except for soluble E-selectin, which was lower after the walnut meal than after the olive oil meal. Jiménez-Gómez *et al.* [115] used meals enriched with walnuts, olive oil and butter in healthy subjects and found similar reductions in postprandial levels of circulating inflammatory biomarkers with the walnut and olive oil meals compared with the SFA-meal. However, these authors also examined mRNA expression of some inflammatory cytokines in circulating blood mononuclear cells and reported that the walnut meal elicited a reduced expression of IL-6 compared to the other two meals [115]. A further sub-study of the PREDIMED trial analyzed both three-month changes in circulating inflammatory biomarkers and in the expression of ligands for inflammatory molecules in circulating monocytes after the study diets, one of which was supplemented with ≈1-oz (30 g) mixed nuts per day [116]. The findings indicate reductions in both soluble ICAM-1 and IL-6 and, importantly, reduced monocyte expression of pro-inflammatory ligands after the walnut-rich mixed nut diet compared with the low-fat diet.

In conclusion, nut consumption appears to have little effect on CRP but it evokes a reduction in concentrations of other inflammatory biomarkers. The gaps in our knowledge of the anti-inflammatory effects of nuts from clinical studies using enriched diets probably stem from the fact that most of them were not designed to evaluate this specific outcome. More recent studies of the effects of nut diets and nut meals on postprandial events and expression of pro-inflammatory molecules in circulating mononuclear cells support a stronger beneficial effect for walnuts while beginning to unravel the molecular bases for the anti-inflammatory effects of nut consumption. Nevertheless, more studies are warranted to conclusively resolve the important question of the antiinflammatory effects of nuts.

#### 4.3.3. Vascular reactivity

Endothelial dysfunction is a critical event in atherogenesis that is implicated both in early disease and in advanced atherosclerosis, where it relates to perfusion abnormalities and the causation of ischemic events [117]. It is characterized by a decreased bioavailability of the endogenous vasodilator NO, synthesized from L-arginine as discussed earlier [29], and increased expression of proinflammatory cytokines and cellular adhesion molecules. Endothelial injury caused by cardiovascular risk factors or atherosclerotic vascular disease reduces NO production and this is followed by arterial wall abnormalities, both functional [inhibition of vasodilatation or paradoxical vasoconstriction] and structural [smooth muscle cell growth and blood cell adhesion] that are responsible for the initiation, development, and progression of atherosclerosis [117].

It has been known for some time that food intake affects vascular reactivity. Short-term feeding studies have consistently shown that diets rich in SFA impair endothelial function [118,119,120]. In addition, a single fatty meal rich in SFA usually is followed by transient endothelial dysfunction in association with elevated triglyceride-rich lipoproteins [121]. Whether acute or chronic, these detrimental effects can be counteracted by the administration of healthy nutrients, such as n-3 PUFA [120], antioxidant vitamins and phenolic compounds [122,123], and L-arginine [124], all of them nut constituents. Thus, it could be predicted that nut consumption could beneficially influence endothelial function.

Walnut diets have been examined *versus* control diets in three randomized crossover studies [74,103,113] by using the standard method for noninvasive assessment of conduit artery endothelial function, flow-mediated dilatation (FMD) in the brachial artery [117]. The first study showed that, by comparison with an isoenergetic Mediterranean diet with similar SFA content, a four-week walnut diet attenuated the endothelial dysfunction associated with hypercholesterolemia [113]. In a follow-up of this trial, it was shown that adding walnuts to a high-fat, high-SFA meal counteracted ensuing postprandial endothelial dysfunction compared to the same meal with added olive oil [103]. The third study, conducted in diabetic patients, compared a walnut diet with an isoenergetic *ad libitum* diet with similar SFA content but without walnuts, each lasting eight weeks, and confirmed that walnuts improve FMD [74]. By analogy with the improvement of endothelial function observed after supplementation of marine n-3 PUFA [118,119,120], this beneficial effect of walnuts may be ascribed in part to their high ALA content. Antioxidants and L-arginine also might have played a role. Of note, supplementing a high-fat meal with ALA from canola oil was also associated with improved postprandial endothelial function in patients with diabetes [125].

Two further studies have assessed the effects of nuts on vascular reactivity [107,126]. A recent study from Turkey reported improved FMD after a diet supplemented with pistachios compared with a healthy diet, but the sequential design of the interventions precludes firm conclusions [126]. Finally, a 12-week parallel design study in patients with metabolic syndrome comparing healthy diets with or without supplementation with 30 g of mixed nuts per day found no between-diet differences in vascular reactivity, as assessed by digital pulse amplitude tonometry [107]. However, it must be noted that this is a non-standard technique to measure endothelial function with much less clinical trial experience than FMD of the brachial artery [117]. 

In summary, there is consisting evidence from two studies that walnut diets improve FMD and from another study that this effect can be observed after a single walnut meal. In support of these observations, Davis *et al.* [127] showed that walnut feeding also reduced the expression of endothelin-1, a potent endothelial activator, in an animal model of accelerated atherosclerosis, and this effect was attributable to the fat component of walnuts. Although there is a paucity of vascular reactivity studies after consumption of diets enriched with nuts other than walnuts, they might be expected to show similar beneficial effects because, with the exception of ALA, particular to walnuts, all nuts contain substantial quantities of bioactive compounds that can favorably influence vascular reactivity. This is a key area for future research.

When endothelial function improves, a lower blood pressure could be predicted. This has not been observed in the usually small-sized clinical studies performed to date, but the larger PREDIMED trial did show significant reductions in both systolic and diastolic blood pressure after the nut-supplemented Mediterranean diet compared with the control diet [80]. A recent report of the PREDIMED trial provides an insight into the possible mechanism of this antihypertensive effect by showing that the nut diet was associated with reduced cholesterol: phospholipid ratios of erythrocyte membranes, which would translate into an increase of membrane fluidity [128]. Possibly future adequately powered studies might uncover a true antihypertensive effect of nut intake. As discussed, there is also insufficient evidence from prospective studies of the relationship between nut consumption and hypertension [50,51]. Further epidemiological and clinical studies on this important topic are clearly needed.

## 5. Safety of Nut Consumption

There are two main concerns regarding the safety of increasing nut consumption: possible weight gain (and worsening metabolic complications of increased adiposity, such as the metabolic syndrome and diabetes) and allergic reactions. While the first can reasonably be dispelled, the second merits attention in particular situations. An additional concern is potential toxicity through contamination of nuts with mycotoxins, particularly aflatoxins, which is a problem that affects agricultural economies and is beyond the scope of this review [reviewed in 129,130].

### 5.1. Body Weight

The common perception that fatty foods provide excess energy and thus promote obesity has had a negative effect on the image of nuts. The question of whether increasing the intake of nuts and therefore calories could lead to unwanted weight gain and related health problems is a critical one. However, as thoroughly reviewed [13,16,26,27], there is considerable scientific evidence indicating that there are no adverse effects of frequent nut consumption on energy balance or body weight. Some studies suggest that nut consumption might even help lose weight.

First, the epidemiological studies that related the frequency of nut consumption with a reduction of incident CHD [1,39,40,41] or diabetes [46] showed a neutral or even inverse association between nut intake and BMI. Recent reports from two large prospective cohorts [131,132] and a cross-sectional study [133] support these findings. In a 28-month prospective study of the SUN cohort conducted in Spain in 8865 university graduates, a significant inverse association between nut consumption and weight gain was reported. Compared with those who never or almost never ate nuts, participants who ate nuts ≥2 times/wk had a 31% lower risk of gaining ≥5 kg during follow-up, while participants who frequently consumed nuts had an average 0.42 kg less weight gain than did those who rarely consumed nuts after multivariate adjustment [131]. The Nurses’ Health Study followed 51,188 women for eight years and showed that women who reported eating nuts ≥2 times/wk had 0.4 kg less mean weight gain than did women who rarely ate nuts, a small but significant difference. The results were similar for tree nuts and peanuts in normal-weight, overweight, and obese participants. In multivariate analyses, greater nut consumption (≥2 times/wk compared with never/almost never) was associated with a slightly lower risk of obesity, with a hazard ratio of 0.77 (CI, 0.57 to 1.02) [132]. In a cross-sectional study of a sample of 847 subjects recruited into the PREDIMED study, nut consumption was inversely associated with adiposity measures (BMI and waist circumference) independently of other lifestyle variables. From regression coefficients of nut intake *versus* adiposity variables, it was predicted that BMI and waist circumference decreased by 0.78 kg/m^2^ and 2.1 cm, respectively, for each serving of 30 g of nuts [133].

Second, the nut intervention trials with outcomes on lipid changes that were carried out in free-living individuals showed no weight gain or a tendency to lose weight in those assigned to nut diets compared with control diets, as reviewed [26,27]. A similar lack of weight gain was documented in the mostly overweight or obese participants in the PREDIMED study who consumed 30 g of mixed nuts per day during three months [80] and in overweight diabetics consuming 30 g of walnuts for six months in a study from Australia [94]. Recent evidence from the PREDIMED study shows a decreased prevalence of the metabolic syndrome, mainly due to reduced visceral adiposity, after intervention for 12 months in participants following a Mediterranean diet supplemented with 30 g of nuts per day [134].

Third, four clinical studies specifically investigated the effects on body weight of supplementing the customary diets of free-living subjects with nuts without constraints on energy balance [135,136,137,138]. In these studies, sizeable quantities of peanuts, almonds or walnuts were provided for daily consumption during periods ranging from eight weeks to six months, without advice on how to include them in their diet. Compared with the corresponding control diet periods, there were insignificant increases or no changes in body weight after the nut diets in all these studies.

Finally, two clinical trials have assessed the efficacy of low-calorie diets with added nuts *versus* conventional low-fat diets for weight loss, and the results showed the nut diets resulting in superior long-term participation and adherence, with consequent improvements in weight loss [139,140].

There are potential mechanisms for the lack of a weight-promoting effect or even a tendency to reduce adiposity of nut consumption in spite of increased energy acquisition. Probably because nuts are fatty foods containing substantial amounts of fiber, their chronic consumption in a free feeding situation causes satiation and elicits a strong dietary compensation, whereby intake of other energy-dense foods is curtailed, which accounts for roughly two thirds of the energy derived from nuts [135,141]. Increased satiation subsequent to nut consumption has been more difficult to detect in acute studies [98,142]. An enhanced thermogenic effect of nut intake was also postulated based on findings from a 19-week feeding trial with peanuts (88 g/day) in healthy subjects, who showed an 11% increase in resting energy expenditure [136]. These findings could not be reproduced in acute studies with walnuts [98,142]. Fat malabsorption, documented during nut diets as increased fecal fat excretion, also can contribute to the lack of weight gain. Increased stool fat losses may be due in part to the high fiber content of nuts [23] or to incomplete digestion of their matrices because the fat of nuts is enclosed within cell membranes, which are not readily available to digestive enzymes [143], an effect that can be compounded by incomplete mastication [144]. At any rate, the satiating effect of nuts with subsequent food compensation appears to be the main reason for their lack of weight-promoting effect.

### 5.2. Allergic Reactions

Nuts are a well known cause of food allergy, with estimated prevalence rates of approximately 1% in the general population [reviewed in 145]. A recent systematic review of population-based studies points to a prevalence of 4.3% when diagnosis is based on food challenge tests and <1% when sensitization is assessed by skin prick test [146]. Allergic reactions to nuts are due to allergenic seed storage proteins that elicit specific IgE antibodies. They affect principally young children and may be particularly severe, even life-threatening. Indeed, fatal anaphylactic reactions following nut ingestion have been documented. Severity of coexisting atopic diseases [asthma, rhinitis, and eczema] predicts which patients are most likely to develop life-threatening allergic reactions to tree nuts and peanuts [147]. A minority of children with peanut allergy developed tolerance with time. Once nut allergy is firmly established, prevention of subsequent episodes, which tend to be clinically worse, includes patient and family education to avoid all types of nuts and be careful of hidden nut products in processed foods. Patients and relatives must be instructed on how to recognize early symptoms of an allergic reaction and how to treat promptly an anaphylactic episode.

## 6. Conclusion

Nuts are energy dense foods rich in bioactive macronutrients, micronutrients and phytochemicals. The unique composition of nuts is critical for their health effects. Indeed, there are consistent evidences from epidemiologic and clinical studies of the beneficial effects of nut consumption on risk of CHD, including sudden cardiac death, as well as on diabetes in women, and on major and emerging cardiovascular risk factors, as summarized in Table 3.

**Table 3:** The effect of nut consumption on cardiovascular risk factors. Summary of scientific evidence.

Variables	Effect	Level of evidence
*Epidemiologic studies*		
Coronary heart disease	Decrease	++
Sudden cardiac death	Decrease	+
Ischemic stroke	No change	+
Heart failure	No change	+
Hypertension	Decrease	+/-
Diabetes	No change/decrease	+/-
Cancer	No change/decrease	+/-
Gallstone disease	Decrease	+
Complications of diverticular disease	Decrease	+
Inflammatory markers	Decrease	+
Body weight	No change/decrease	++
*Clinical studies*		
Blood cholesterol	Decrease	++
Insulin sensitivity	No change/increase	+/-
Blood pressure	Decrease	+
Oxidation	No change/decrease	+/-
Inflammation	No change/decrease	+/-
Vascular reactivity	Increase	+
Body weight	No change	++
Visceral adiposity	Decrease	+

+/-, equivocal evidence; +, limited evidence from few studies; ++, consisting evidence in several studies.

The evidence to date is convincing that including nuts in a healthy dietary pattern will extend the cardioprotective effects beyond those attributable to the components of any healthy diet exclusive of nuts. Importantly, these effects take place without undue weight gain, or even with reduced adiposity, and target multiple cardiovascular risk factors and mechanisms, which help explain why nuts so potently reduce the risk for CHD. There is also emerging evidence from acute studies that single meals enriched with nuts can have a beneficial impact on postprandial events related to atherogenesis, such as glucose and triglyceride raises, inflammation, and endothelial activation. Understanding the underlying biological mechanisms of the effects of nuts on mediators of CHD, obesity, metabolic syndrome, diabetes, and cancer should help in the design of diets that include nuts to maximally reduce chronic disease risk. The vegetarian, Mediterranean, and many Asian diets are traditional plant-based dietary patterns that include nuts and are reputed for their beneficial effects on health.

A healthy dietary pattern is high in vegetables, fruits, legumes, nuts, whole grains, and lean protein sources and low-fat dairy products [10]. Nuts are a popular and important source of unsaturated fat and high-quality vegetable protein in vegetarian diets, where they rank high on the list of foods most frequently consumed, above meat substitutes [9,148]. The optimal nutrient composition of nuts and the impressive evidence gained from epidemiologic and clinical studies on their health benefits indicates that they are an indispensable contribution to a well-balanced vegetarian diet. Also, knowledge has accumulated that dietary patterns close to the Mediterranean diet, in which nuts are a key food item, are associated with many beneficial health outcomes [16,149]. Indeed both exposure to the Mediterranean diet and frequency of nut consumption are among the dietary factors with stronger evidences for a causal link with CHD prevention [150]. Ongoing research like the large randomized PREDIMED trial, wherein one daily serving of mixed nuts within the context of the Mediterranean diet is provided to participants at high cardiovascular risk in one arm of this six-year study [80], might eventually settle the critical issues of whether, in comparison with a healthy control diet without nuts, a healthy diet supplemented with one daily serving of nuts prevents cardiovascular events and development of other prevalent chronic disorders, including diabetes, cancer and neurodegenerative diseases.

## Conflict of Interest

The author has received research funding from the California Walnut Commission, Sacramento, CA and is a non paid member of its Scientific Advisory Committee.
